# Supplementary material for: Genetic changes and testing associated with childhood glaucoma: A systematic review
Source: PLoS One. 2024 Feb 22;19(2):e0298883. doi: 10.1371/journal.pone.0298883 (PMC10883561; doi:10.1371/journal.pone.0298883)
Supplement: S1 Protocol — (PDF) [file pone.0298883.s002.pdf]

Genetic testing and identified variants in childhood glaucoma: a systematic review

To enable PROSPERO to focus on COVID-19 submissions, this registration record has undergone basic automated checks for eligibility and is published exactly as submitted. PROSPERO has never provided peer review, and usual checking by the PROSPERO team does not endorse content. Therefore, automatically published records should be treated as any other PROSPERO registration. Further detail is provided here.

Citation

Anika Kumar, Julius Oatts. Genetic testing and identified variants in childhood glaucoma: a systematic review. PROSPERO 2023 CRD42023400467 Available from:
https://www.crd.york.ac.uk/prospero/display_record.php?ID=CRD42023400467

Review question

To describe genetic testing strategies and genetic changes reported in the setting of childhood glaucoma.

Searches

PubMed, Embase, Cochrane Library

Search date: 2/20/23

No language or publication date restrictions

Types of study to be included

• Inclusion criteria

- Study design: prospective cohort study, retrospective cohort study, case report or case series

- Patients 0-18 years old

- Evaluation/discussion of one or more genes and/or a specific genetic test/panel

• Exclusion criteria

- Unrelated/irrelevant

- Review articles, letters, case reports, abstract-only papers, unavailable full text

Condition or domain being studied

Glaucoma affecting patients less than 18 years old

Participants/population

- Patients 0-18 years old with diagnosis of glaucoma of any etiology

Intervention(s), exposure(s)

Genetic changes implicated in childhood glaucoma

Genetic tests associated with childhood glaucoma

Comparator(s)/control

Not applicable

Context

Glaucoma in rare condition in children which can cause irreversible blindness if not diagnosed and treated in a time sensitive manner. Many forms of childhood glaucoma including primary congenital glaucoma, juvenile open angle glaucoma, and glaucoma associated with non-acquired ocular or systemic disease are thought to be associated with underlying genetic changes. While several genes have been identified, genetic testing can be inconsistent or inconclusive.

Testing for genetic markers associated with childhood glaucoma can better inform the development of individualized treatment and surveillance plans. Although many genes have been implicated in the development of childhood glaucoma, currently, no clinical practice guidelines exist outlining protocols for genetic testing. This may be driven by the relative nascency of the field of childhood glaucoma genetics that has not yet resulted in enough centralized high quality evidence to influence standard clinical practice. This study aims to summarize the current body of evidence evaluating genetic testing and genetic changes described in childhood glaucoma.

Main outcome(s)

Primary outcome of interest: Genetic testing strategies in childhood glaucoma

• Percent yield of different genetic testing strategies

• Summary of current described genetic changes in childhood glaucoma and modality of diagnosis (i.e.: panel, whole exome sequencing)

Secondary outcome of interest: genotype/phenotype correlation

Measures of effect

percent yield of different genetic testing strategies

Additional outcome(s)

none

Measures of effect

none

Data extraction (selection and coding)

Study Selection

• Querying above databases and additional sources to gather all relevant articles

• Removing duplicates

• Screening based on inclusion and exclusion criteria by one person

• Full text review and extraction of relevant data into Excel by one person

• Validation of screening and extraction on a random subset of 10% of studies by second person

Data Extraction

• Year of publication of study

• Study design

- 1 = prospective cohort

- 2 = retrospective cohort

- 3 = case report or case series

- 4 = other

• Sample size

• Study population information

- Mean [SD] age

- Sex

- Family history of glaucoma

• Gene(s) or genetic test(s) identified

• Type of gene

- Gene that increases risk

- Gene that directly causes glaucoma

- Genes of uncertain significance

• More details about genotypic-phenotypic relationship

• Inheritance pattern of gene

• Type of glaucoma associated with gene

- 1 = primary congenital glaucoma

- 2 = juvenile open-angle glaucoma

- 3 = glaucoma following cataract surgery

- 4 = glaucoma associated with non-acquired ocular or systemic disease

Risk of bias (quality) assessment

Risk of bias will be performed independently by 2 investigators using the ROBINS-I tool for non-randomized controlled trials. Disagreements will be adjudicated by a third party.

Strategy for data synthesis

A qualitative synthesis of individual studies will be performed to discuss individual findings per the variables extracted. No meta-analysis is planned.

Analysis of subgroups or subsets

If data allows, specific discussion of findings by various etiologies of glaucoma will be included.

Contact details for further information

Anika Kumar

anika.kumar@ucsf.edu

Organisational affiliation of the review

University of California, San Francisco

Review team members and their organisational affiliations

Ms Anika Kumar. University of California, San Francisco

Dr Julius Oatts. University of California, San Francisco

Collaborators

Dr Ying Han. University of California, San Francisco

Type and method of review

Systematic review

Anticipated or actual start date

20 February 2023

Anticipated completion date

24 April 2023

Funding sources/sponsors

none

Conflicts of interest

None known

Language

English

Country

United States of America

Stage of review

Review Ongoing

Subject index terms status

Subject indexing assigned by CRD

Subject index terms

Genetic Testing; Humans; Hydrophthalmos

Date of registration in PROSPERO

28 February 2023

Date of first submission

17 February 2023

Stage of review at time of this submission

The review has not started

**Stage**	**Started**	**Completed**
Preliminary searches	No	No
Piloting of the study selection process	No	No
Formal screening of search results against eligibility criteria	No	No
Data extraction	No	No
Risk of bias (quality) assessment	No	No
Data analysis	No	No

The record owner confirms that the information they have supplied for this submission is accurate and complete and they understand that deliberate provision of inaccurate information or omission of data may be construed as scientific misconduct.

The record owner confirms that they will update the status of the review when it is completed and will add publication details in due course.

Versions

28 February 2023

28 February 2023
